# Impact of coronary calcification assessed by coronary CT angiography on treatment decision in patients with three-vessel CAD: insights from SYNTAX III trial

**DOI:** 10.1093/icvts/ivab249

**Published:** 2021-09-20

**Authors:** Daniele Andreini, Kuniaki Takahashi, Saima Mushtaq, Edoardo Conte, Rodrigo Modolo, Jeroen Sonck, Johan De Mey, Paolo Ravagnani, Danny Schoors, Francesco Maisano, Philipp Kaufmann, Wietze Lindeboom, Marie-angele Morel, Torsten Doenst, Ulf Teichgräber, Gianluca Pontone, Giulio Pompilio, Antonio Bartorelli, Yoshinobu Onuma, Patrick W Serruys, the Syntax III Revolution Investigators

**Affiliations:** Department of Cardiovascular Imaging, Centro Cardiologico Monzino, IRCCS, Milan, Italy; Department of Clinical Sciences and Community Health, University of Milan, Milan, Italy; Department of Cardiology, Amsterdam University Medical Center, Amsterdam, Netherlands; Department of Cardiovascular Imaging, Centro Cardiologico Monzino, IRCCS, Milan, Italy; Department of Cardiovascular Imaging, Centro Cardiologico Monzino, IRCCS, Milan, Italy; Department of Cardiology, Amsterdam University Medical Center, Amsterdam, Netherlands; Cardiology Division, Department of Internal Medicine, Hospital de Clinicas, University of Campinas, Campinas, São Paulo, Brazil; Cardiovascular Center Aalst, OLV Hospital, Aalst, Belgium; Universitair Ziekenhuis Brussel, Vrije Universiteit Brussel, Brussel, Belgium; Department of Cardiovascular Imaging, Centro Cardiologico Monzino, IRCCS, Milan, Italy; University of Zurich, Zurich, Switzerland; University of Zurich, Zurich, Switzerland; University of Zurich, Zurich, Switzerland; Cardialysis BV, Rotterdam, Netherlands; Cardialysis BV, Rotterdam, Netherlands; Jena University Hospital, Friedrich-Schiller-University of Jena, Jena, Germany; Jena University Hospital, Friedrich-Schiller-University of Jena, Jena, Germany; Department of Cardiovascular Imaging, Centro Cardiologico Monzino, IRCCS, Milan, Italy; Department of Cardiovascular Imaging, Centro Cardiologico Monzino, IRCCS, Milan, Italy; Department of Clinical Sciences and Community Health, University of Milan, Milan, Italy; Department of Cardiovascular Imaging, Centro Cardiologico Monzino, IRCCS, Milan, Italy; Department of Biomedical and Clinical Sciences “Luigi Sacco”, University of Milan, Milan, Italy; Thoraxcenter, Erasmus MC, Rotterdam, Netherlands; Department of Cardiology, Royal Brompton and Harefield Hospitals, Imperial College London, London, UK

**Keywords:** Coronary calcification, Coronary computed tomography angiography, Heart team

## Abstract

**OBJECTIVES:**

The aim of this study was to determine Syntax scores based on coronary computed tomography angiography (CCTA) and invasive coronary angiography (ICA) and to assess whether heavy coronary calcification significantly limits the CCTA evaluation and the impact of severe calcification on heart team’s treatment decision and procedural planning in patients with three-vessel coronary artery disease (CAD) with or without left main disease.

**METHODS:**

SYNTAX III was a multicentre, international study that included patients with three-vessel CAD with or without left main disease. The heart teams were randomized to either assess coronary arteries with coronary CCTA or ICA. We stratified the patients based on the presence of at least 1 lesion with heavy calcification defined as arc of calcium >180° within the lesion using CCTA. Agreement on the anatomical SYNTAX score and treatment decision was compared between patients with and without heavy calcifications.

**RESULTS:**

Overall, 222 patients with available CCTA and ICA were included in this trial subanalysis (104 with heavy calcification, 118 without heavy calcification). The mean difference in the anatomical SYNTAX score (CCTA derived—ICA derived) was lower in patients without heavy calcifications [mean (−1.96 SD; +1.96 SD) = 1.5 (−19.3; 22.4) vs 5.9 (−17.5; +29.3), *P* = 0.004]. The agreement on treatment decision did not differ between patients with (Cohen’s kappa 0.79) or without coronary calcifications (Cohen’s kappa 0.84). The agreement on the treatment planning did not differ between patients with (concordance 80.3%) or without coronary calcifications (concordance 82.8%).

**CONCLUSIONS:**

An overall good correlation between CCTA- and ICA-derived Syntax score was found. The presence of heavy coronary calcification moderately influenced the agreement between CCTA and ICA on the anatomical SYNTAX score. However, agreement on the treatment decision and planning was high and irrespective of the presence of calcified lesions.

**Figure:**
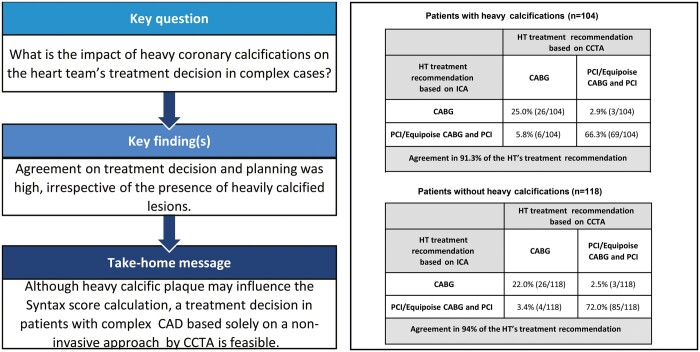

## INTRODUCTION

Severe calcifications of vessel wall and atherosclerotic plaque hamper visual assessment of coronary arteries with coronary computed tomography angiography (CCTA) due to blooming artefacts. This drawback may explain the discrepancy that has been observed between non-invasive and invasive luminal evaluation [1]. Although scanners with improved spatial resolution and/or dual-energy technology may overcome most of the beam-hardening artefacts [2, 3], assessment of coronary arteries with a high calcific burden is still a weakness of this non-invasive imaging modality. Moreover, severe calcifications, which are easily detectable with CCTA, may influence treatment decision-making and planning particularly in patients with multivessel coronary artery disease (CAD). The SYNTAX III Revolution trial showed that the treatment decision-making based on CCTA in patients with three-vessel CAD is in high agreement (Cohen’s kappa 0.82) with the decision derived from invasive coronary angiography (ICA) [4]. However, the influence of coronary calcifications on treatment decision-making and selection of vessels to be revascularized remains to be investigated. Thus, the present study sought to determine the impact of heavy coronary calcifications on heart team’s treatment decision and procedural planning in patients with three-vessel CAD.

## METHODS

### Study design

Date and number of the IRB approval are as follows: 05 August 2016 and CCM441 (trial registration number: NCT02813473).

The present study reports a predefined subanalysis of the SYNTAX III REVOLUTION trial. The design of the SYNTAX III REVOLUTION trial has been reported previously [5]. The trial was an international, multicentre study in which 2 heart teams (HTs) composed of an interventional cardiologist, a cardiac surgeon and a cardiac radiologist were randomized to assess and characterize CAD with either CCTA or ICA in patients with three-vessel CAD with or without left main coronary artery involvement, although all patients underwent both CCTA and ICA. The results of the primary end point represented by the agreement between the 2 HTs on the revascularization strategy have been recently published and showed a very high agreement [Cohen’s kappa 0.82; 95% confidence interval (CI) 0.74–0.91], suggesting the potential feasibility of a treatment decision-making and planning based solely on this non-invasive imaging modality [4]. For the present analysis, patients were stratified based on the presence of at least 1 coronary lesion with heavy calcification defined as an arc of calcium >180° within the lesion using CCTA. Agreement on the anatomical SYNTAX score and treatment decision was compared between patients with and without heavy calcifications. The study was approved by the investigational review board and ethics committee at each participating centre.

### Enrolment and randomization

Patients with three-vessel CAD diagnosed with either CCTA or ICA and candidates for either percutaneous coronary intervention (PCI) or CABG were assessed for eligibility. Patients were consented to undergo CCTA using a whole-heart coverage, high-definition CT scanner (Revolution CT, GE Healthcare, Chicago, IL, USA) and to participate in a randomized trial of decision-making between PCI and CABG performed by the local heart team and relying on alternative imaging techniques. Two HTs were randomized to either assess the coronary anatomy with CCTA or ICA in addition to the patient’s clinical information. The members of the HTs involved in the original study were totally blinded to the CCTA and ICA findings before to assess the imaging modality assigned to their specific heart team (CCTA or ICA). Each HT calculated the anatomical SYNTAX score based only on their allocated imaging modality and subsequently integrated the clinical information to compute the SYNTAX score II providing a treatment recommendation, i.e. CABG, PCI or equipoise between CABG and PCI. In particular, the HT’s treatment recommendation led to 1 of 3 decisions according to the SYNTAX score II and other anatomical and clinical information including coronary calcification: (i) CABG only, patients should be treated by CABG due to a higher 4-year mortality with PCI; (ii) PCI only, patients should be treated by PCI due to a higher 4-year mortality with CABG; and (iii) equipoise between CABG and PCI, patients could be treated by either approach, considering that the 4-year mortality prediction is similar between them. Any anatomical SYNTAX score was eligible for screening and patients with an anatomical SYNTAX score >33 were not excluded. Patients with prior revascularization were excluded. Complete details of the inclusion and exclusion criteria have been previously described [4, 5].

### Image acquisition and analysis

CCTA was performed using the GE Revolution CT scanner with a spatial resolution of 230 μm along the *X*–*Y* planes, a *Z*-axis resolution of 625 μm, a rotational speed of 0.28 s and a *Z*-plane coverage of 16 cm enabling to image the heart in 1 heartbeat [6]. The imaging acquisition guidelines are detailed in the Supplementary Material, Table S1. The protocol mandated the use of nitrates prior to CCTA acquisition and beta-blockers in cases of heart rate higher than 65 bpm. The 2 local heart teams signed off their decision on the choice of revascularization mode based on the anatomical assessment alone. The anatomic SYNTAX scores were also calculated by an independent core laboratory (Cardialysis BV, Rotterdam, The Netherlands) and were made available to each heart team for consultation.

### Objectives

The primary objective of the present study was to determine the difference, assessed by Cohen’s kappa, in treatment recommendation, based on either CCTA or ICA, between 2 HTs in patients with or without heavy calcifications. The secondary objective was to determine the difference in treatment planning, defined as the coronary segments to be revascularized, between 2 HTs in patients with or without heavy calcifications.

### Statistical analysis

The HT’s treatment recommendation led to 1 of 3 decisions according to the SYNTAX score II: (i) CABG only, patients should be treated by CABG due to a higher 4-year mortality with PCI; (ii) PCI only, patients should be treated by PCI due to a higher 4-year mortality with CABG; and (iii) equipoise between CABG and PCI, patients could be treated by either approach, considering that the 4-year mortality prediction is similar between them. The power calculation of the sample size of the SYNTAX III REVOLUTION trial has been previously described [5]. However, for the present subanalysis, 2 subsamples of 118 and 104 subjects provided 80% power to deem as significant (alpha = 0.05) a difference of at least 0.25 between the 2 kappa values (for instance, 0.89 vs 0.64). Continuous variables were compared with the Student’s *t*-test for normally distributed or the Wilcoxon rank-sum test for non-normally distributed data, respectively. Differences in categorical variables were assessed with the *χ*^2^ test or, in case of values below 5 in any cells of contingency tables, Fisher’s exact test. These criteria were prespecified. Agreement between SYNTAX II recommendation strategy derived from ICA only versus derived from ICA and CCTA was assessed with the concordance coefficient of kappa. Briefly, the Cohen's kappa coefficient (*K*) is used to measure inter-rater reliability (and also intrarater reliability) for qualitative (categorical) items. The agreement on the SYNTAX score between the 2 imaging modalities was assessed by the Pearson correlation and Bland–Altman or Passing Bablok method [7, 8]. A two-sided *P*-value of 0.05 or less was considered to indicate statistical significance. All statistical analyses were performed with the use of SAS software, version 9.4 (SAS Institute).

## RESULTS

**Figure 1:**
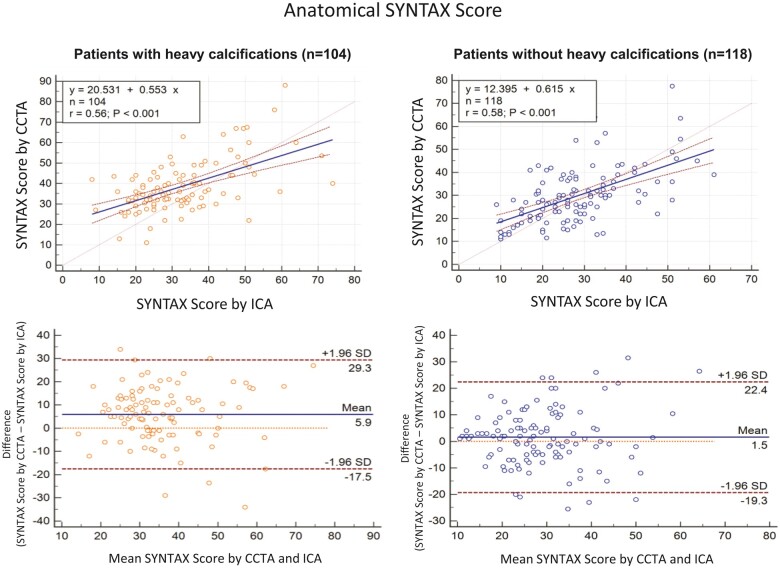
Anatomical SYNTAX score. Correlations (upper panels) and differences (bottom panels) between anatomical Syntax score derived from CCTA and ICA in patients with (left panels) and without (right panels) heavy coronary calcifications. CCTA: coronary computed tomography angiography; ICA: invasive coronary angiography.

**Figure 2:**
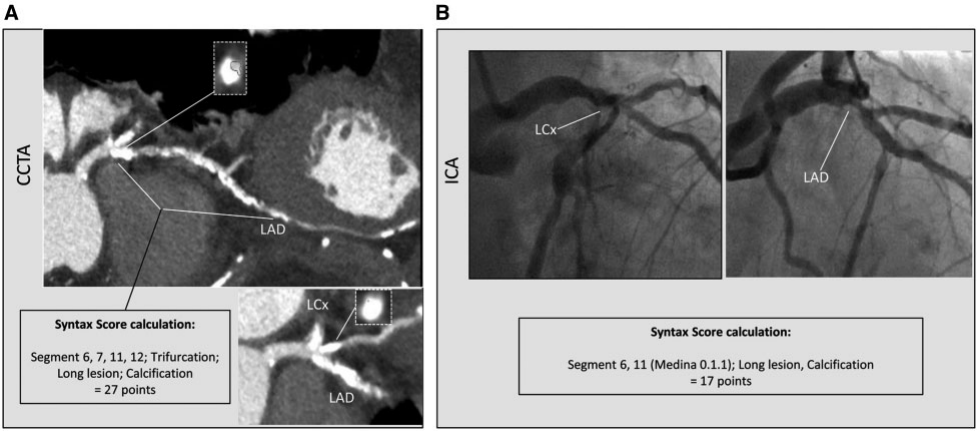
Calcified coronary lesion with Syntax score discrepancy. A case of a heavily calcified lesion leading to discrepancy between CCTA-derived (**A**) and ICA-derived (**B**) SYNTAX scores. CCTA: coronary computed tomography angiography; ICA: invasive coronary angiography; LAD: left anterior descending; LCX: left circumflex.

**Figure 3:**
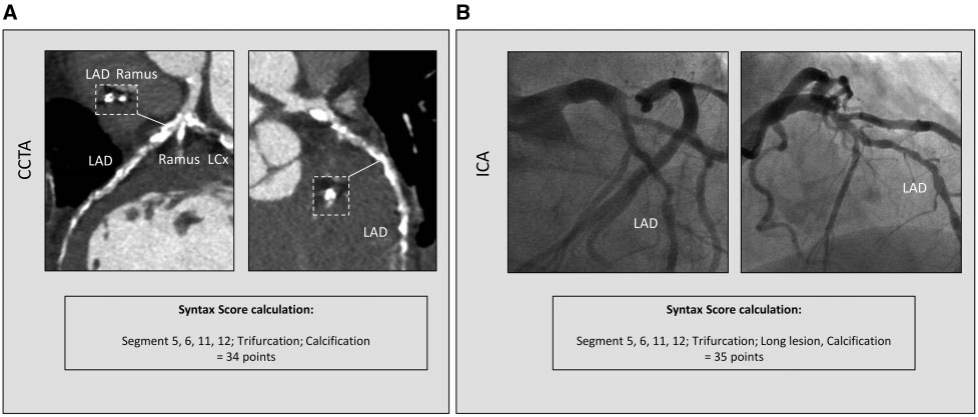
Calcified coronary lesion without Syntax score discrepancy. A case of a heavily calcified lesion not affecting the calculation of CCTA-derived (**A**) and ICA-derived (**B**) SYNTAX scores. CCTA: coronary computed tomography angiography; ICA: invasive coronary angiography; LAD: left anterior descending; LCX: left circumflex.

From 29 June 2016 to 8 February 2018, 223 patients with three-vessel CAD were enrolled in 6 centres from 5 European countries. Baseline clinical characteristics and CCTA acquisition data of the entire SYNTAX III population are shown in Supplementary Material, Table S2. CCTA assessment was feasible in 222/223 (99%) patients. Baseline clinical characteristics and CCTA acquisition data in patients with (*n* = 104) or without (*n* = 118) heavy calcifications are reported in Table 1. Patients with heavy calcification were significantly older (mean age 69.5 ± 7.9 vs 65.9 ± 9.4, *P* = 0.002) and more often had diabetes (46.2% vs 30.5%, *P* = 0.016). No other significant differences were found between the 2 groups. As previously reported, in the entire population, the mean anatomical SYNTAX score derived from CCTA was 33.9 ± 13.0, whereas that derived from ICA was 30.3 ± 12.2, with a mean difference between them of 3.58 (−1.96; +1.96 SD = −18.8; 25.9, respectively) and a correlation coefficient of 0.59 (*P* < 0.0001) [4]. In patients with heavy calcification, the mean computed tomography angiography (CTA)-derived anatomical SYNTAX score was 38.6 ± 12.7, whereas the mean SYNTAX score derived from ICA was 32.7 ± 12.9, with a mean difference between them of 5.9 (−1.96; +1.96 SD = −17.5; 29.3, respectively) and a correlation coefficient of 0.56 (*P* < 0.001) (*y* = 0.553 × *x* + 20.531) (Fig. 1). In patients without heavy calcification, the mean CTA-derived anatomical SYNTAX score was 29.7 ± 11.9, whereas that derived from ICA was 28.2 ± 11.3, with a mean difference between them of 1.5 (−1.96; +1.96 SD = −19.3; 22.4) and a correlation coefficient of 0.58 (*P* < 0.001) (*y* = 0.615 × *x* + 12.395) (Fig. 1). The mean difference in the anatomical SYNTAX score was significantly lower in patients without heavy calcifications compared to those with heavy calcifications (*P* = 0.004). CCTA-Syntax score and ICA-Syntax score were significantly different only in patients with heavy calcifications, as shown in Table 2. Figures 2 and 3 show 2 case examples of heavily calcified coronary lesions with (Fig. 2) and without (Fig. 3) discrepancy between the anatomical SYNTAX scores derived from CCTA and ICA.

**Table 1:** Baseline clinical characteristics of patients with and without heavy calcification

Characteristics	Patients with heavy calcifications (*n* = 104)	Patients without heavy calcifications (*n* = 118)	*P*-value
Demographics			
Age (years), mean ± SD	69.5 ± 7.9	65.9 ± 9.4	0.002
Male gender, % (*n*)	86.5 (90/104)	82.2 (97/118)	0.376
CAD risk factors, % (*n*)			
Current smoking	17.3 (17/98)	27.4 (31/113)	0.081
Diabetes mellitus	46.2 (48/104)	30.5 (36/118)	0.016
Treatment for diabetes			
Insulin	13.5 (14/104)	7.6 (9/118)	0.109
Medication	29.8 (31/104)	22 (26/118)	
Diet	1.9 (2/104)	0 (0/118)	
Hypertension	76.9 (80/104)	72.9 (86/118)	0.489
Hyperlipidaemia	70.9 (73/104)	69 (80/116)	0.759
Family history of CAD	34.9 (30/86)	35.6 (36/101)	0.914
Medical history, % (*n*)			
Previous stroke	8.7 (9/104)	7.6 (9/118)	0.78
Previous myocardial infarction	2 (2/102)	0 (0/118)	0.128
COPD	13.5 (14/104)	12.7 (15/118)	0.869
PVD	20.2 (21/104)	15.3 (18/118)	0.335
Clinical presentation			0.898
Silent ischaemia, % (*n*)	43.3 (45/104)	41.5 (49/118)	
Unstable angina, % (*n*)	9.6 (10/104)	8.5 (10/118)	
Stable angina, % (*n*)	47.1 (49/104)	50 (59/118)	
BMI (kg/m^2^), mean ± SD	26.7 ± 3.8	26.4 ± 3.7	0.545
Creatinine clearance (ml/min), mean ± SD	79.6 ± 26.9	83.4 ± 28.1	0.298
LVEF (%), mean ± SD	54.3 ± 11.2	54.9 ± 10.9	0.706
CCTA data, mean ± SD			
Heart rate during CCTA acquisition	62.7 ± 8.2	61.7 ± 8.9	0.401
CCTA effective dose (mSv)	4.9 ± 2.6	5.2 ± 3.4	0.473

BMI: body mass index; CAD: coronary artery disease; CCTA: coronary computed tomography angiography; COPD: chronic obstructive pulmonary disease; LVEF: left ventricle ejection fraction; mSv: milliSievert; PVD: peripheral vascular disease; SD: standard deviation.

**Table 2:** CCTA-Syntax score and ICA-Syntax scores in patients with or without heavy calcification

	CCTA-Syntax score	ICA-Syntax score	*P*-value
Patients with heavy calcification	38.6 ± 12.7	32.7 ± 12.9	<0.001
Patients without heavy calcification	29.7 ± 11.9	28.2 ± 11.3	0.119

CCTA: coronary computed tomography angiography; ICA: invasive coronary angiography.

### Primary end point: differences in treatment decision

As previously reported, in the whole population the agreement concerning HT’s treatment recommendation between CCTA and ICA was very high (93%), according to the statistical nomenclature of Cohen’s kappa, with a coefficient of 0.82 (95% CI 0.73–0.90) [4]. In patients with heavy calcification, the agreement concerning HT’s treatment recommendation between the 2 imaging modalities was also very high (91.3%), with a kappa coefficient of 0.79 (95% CI 0.66–0.92). In patients without heavy calcification, similar results in terms of agreement in the HT’s treatment recommendation (94%) and related Cohen’s kappa coefficient (0.84, 95% CI 0.73–0.96) were found.

### Secondary end point: differences in procedural planning

Overall, the heart teams agreed on the coronary vessels to be revascularized in 81.1% of the cases [4]. The agreement on treatment planning did not differ between patients with heavy vessel calcifications [concordance ranged between 94.2% (left anterior descending artery) and 66.3% (left circumflex artery), overall vessels’ concordance of 80.3% (501/624 vessels)] (Table 3) and those without calcifications [concordance ranged between 94.9% (left anterior descending artery) and 64.4% (left circumflex artery), overall vessels’ concordance of 82.8% (586/708 vessels)] (Table 4).

**Table 3:** Agreement between 2 heart teams on the treatment planning, defined as the coronary vessels to be revascularized

Patients without heavy calcification		
LAD (concordance 94.9%)	Not intended to be treated	Intended to be treated
Not intended to be treated	0.8% (1/118)	4.2% (5/118)
Intended to be treated	0.8% (1/118)	94.1% (111/118)
LCx (concordance 64.4%)	Not intended to be treated	Intended to be treated
Not intended to be treated	41.5% (49/118)	12.7% (15/118)
Intended to be treated	22.9% (27/118)	22.9% (27/118)
Diagonals (concordance 90.7%)	Not intended to be treated	Intended to be treated
Not intended to be treated	66.9% (79/118)	8.5% (10/118)
Intended to be treated	14.4% (17/118)	10.2% (12/118)
RCA (concordance 90.7%)	Not intended to be treated	Intended to be treated
Not intended to be treated	14.4% (17/118)	6.8% (8/118)
Intended to be treated	2.5% (3/118)	76.3% (90/118)
LM (concordance 77.1%)	Not intended to be treated	Intended to be treated
Not intended to be treated	17.8% (21/118)	16.1% (19/118)
Intended to be treated	6.8% (8/118)	59.3% (70/118)
Ramus (concordance 92.4%)	Not intended to be treated	Intended to be treated
Not intended to be treated	83.9% (99/118)	5.1% (6/118)
Intended to be treated	2.5% (3/118)	8.5% (10/118)

Angio first team: first decision based on ICA (columns) versus CCTA first team: first decision based on CCTA only (rows). All vessels concordance 82.8% (586/708 vessels).

CCTA: coronary computed tomography angiography; ICA: invasive coronary angiography; LAD: left anterior descending; LCx: left circumflex; LM: left main; Ramus: intermediate ramus; RCA: right coronary artery.

**Table 4:** Agreement between 2 heart teams on the treatment planning, defined as the coronary vessels to be revascularized

Patients with heavy calcification		
LAD (concordance 94.2%)	Not intended to be treated	Intended to be treated
Not intended to be treated	1.0% (1/104)	4.8% (5/104)
Intended to be treated	1.0% (1/104)	93.3% (97/104)
LCx (concordance 66.3%)	Not intended to be treated	Intended to be treated
Not intended to be treated	53.8% (56/104)	11.5% (12/104)
Intended to be treated	22.1% (23/104)	12.5% (13/104)
Diagonals (concordance 66.3%)	Not intended to be treated	Intended to be treated
Not intended to be treated	50.0% (52/104)	21.2% (22/104)
Intended to be treated	12.5% (13/104)	16.3% (17/104)
RCA (concordance 87.5%)	Not intended to be treated	Intended to be treated
Not intended to be treated	4.8% (5/104)	11.5% (12/104)
Intended to be treated	1.0% (1/104)	82.7% (86/104)
LM (concordance 79.8%)	Not intended to be treated	Intended to be treated
Not intended to be treated	1.9% (2/104)	16.3% (17/104)
Intended to be treated	3.8% (4/104)	77.9% (81/104)
Ramus (concordance 87.5%)	Not intended to be treated	Intended to be treated
Not intended to be treated	78.8% (82/104)	4.8% (5/104)
Intended to be treated	7.7% (8/104)	8.7% (9/104)

Angio first team: first decision based on ICA (columns) versus CCTA first team: first decision based on CCTA only (rows). All vessels concordance 80.3% (501/624 vessels).

CCTA: coronary computed tomography angiography; ICA: invasive coronary angiography; LAD: left anterior descending; LCx: left circumflex; LM: left main; Ramus: intermediate ramus; RCA: right coronary artery.

### SYNTAX score II and heart team’s treatment recommendation based on computed tomography angiography-Syntax score and invasive coronary angiography-Syntax score

Differences between patients with and without heavy vessel calcifications regarding the final revascularization strategy based on the Syntax II scores and the clinical evaluation are reported in Table 5.

**Table 5:** SYNTAX score II and heart team’s treatment recommendation based on CTA-Syntax score and ICA-Syntax score

	Patients with heavy calcification	Patients without heavy calcification	*P*-value
CTA-Syntax score, mean ± SD	38.6 ± 12.7	29.7 ± 11.9	<0.001
Syntax score II (PCI), mean ± SD	39.4 ± 11.0	34.6 ± 10.5	0.001
Syntax score II (CABG), mean ± SD	34.4 ± 11.4	30.4 ± 11.6	0.010
Heart team’s treatment recommendation, % (*n*)			<0.001
CABG	65.4 (68/104)	42.4 (50/118)	
Equipoise: final decision CABG	24.0 (25/104)	22.0 (26/118)	
Equipoise: final decision PCI	6.7 (7/104)	17.0 (20/118)	
PCI	3.9 (4/104)	18.6 (22/118)	
ICA-Syntax score, mean ± SD	32.7 ± 12.9	28.2 ± 11.3	0.005
Syntax score II (PCI), mean ± SD	37.8 ± 10.9	34.3 ± 10.5	0.015
Syntax score II (CABG), mean ± SD	34.4 ± 11.5	30.1 ± 11.5	0.006
Heart team’s treatment recommendation, % (*n*)			0.004
CABG	56.7 (59/104)	38.1 (45/118)	
Equipoise: final decision CABG	21.2 (22/104)	18.6 (22/118)	
Equipoise: final decision PCI	5.8 (6/104)	18.6 (22/118)	
PCI	16.4 (17/104)	24.6 (29/118)	

CABG: coronary artery bypass graft; CTA: computed tomography angiography; ICA: invasive coronary angiography; PCI: percutaneous coronary intervention.

## DISCUSSION

The main findings of the present study can be summarized as follows: (i) as expected, heavy coronary calcifications affected the capability of CCTA to accurately assess the anatomical SYNTAX score. Indeed, we found a significantly higher difference between the CCTA-derived anatomical SYNTAX score compared with that derived from ICA in patients with heavy calcifications versus those without heavy calcifications (difference of 5.9 vs 1.5 points, respectively, *P* = 0.004); (ii) despite the discrepancy in the anatomical SYNTAX score assessment, the agreement on the HT’s treatment decision did not differ in patients with (Cohen’s kappa 0.79) or without heavy calcifications (Cohen’s kappa 0.84); and (iii) the agreement on the treatment planning, defined as the coronary vessels to be revascularized, was high and did not differ between patients with (overall vessels’ concordance 80.3%) or without heavy calcifications (overall vessels’ concordance 82.8%).

The results of the SYNTAX III REVOLUTION trial suggest the potential feasibility of a treatment decision-making and planning in patients with complex and multivessel CAD based solely on a non-invasive approach represented by CCTA. However, some concerns remain on the capability of CCTA to serve as decision-making tool in patients with a high calcific coronary burden, a frequent condition in complex and diffuse CAD, particularly in elderly and diabetic patients. Indeed, CCTA images are less accurate and interpretable in these settings, often leading to the overestimation of lesion severity with a negative impact on the specificity and accuracy of the method [1, 9]. On the other hand, in comparison with ICA, CCTA offers not only the possibility for assessing calcium distribution but also a roadmap along coronary arteries. Moreover, compared to invasive optical coherence tomography, CCTA provides similar information, despite a systematic overestimation of calcific plaque volume [10]. In our subanalysis of the SYNTAX III REVOLUTION trial, patients with heavily calcified lesions were significantly older (mean age 69.5 ± 7.9 vs 65.9 ± 9.4) and more often had diabetes (46.2% vs 30.5%). Of note, despite these unfavourable clinical conditions and a higher SYNTAX score (38.6 ± 12.7), the correlation between CCTA-derived and ICA-derived SYNTAX scores was also high in patients with calcifications (*r* = 0.56, *P* < 0.001) and similar to that obtained in patients without calcifications (*r* = 0.58, *P* < 0.001). Accordingly, the main finding of this study was that the presence of heavily calcified lesions did not affect the capability of CCTA to guide treatment decision-making (Cohen’s *K* of 0.79 for CCTA versus ICA) and treatment planning, with a concordance that was 80.3% for all vessels. These remarkable results are likely due, at least in part, to the innovative technology with improved spatial resolution (0.23 mm) of the CT scanner used in this trial. Indeed, this scanner demonstrated to be more accurate in patients with high pretest likelihood of CAD and atherosclerotic burden [3]. Another potential reason why the overall quality of CCTA images was high is the low heart rate achieved during scanning (62.7 ± 8.2 and 61.7 ± 8.9 bpm in patients with and without coronary calcifications, respectively) due to a strict adherence to the protocol acquisition guidelines. Indeed, the protocol recommended heart rate modulation by beta-blockers in patients with >60 bpm during breath holding. Although the overall positive findings, some concerns remain on the relatively low concordance between heart team’s treatment recommendation based on the 2 imaging modalities for left circumflex artery (concordance in about the two-third of patients in both groups) and diagonal branches (concordance in 66% of patients in the group with heavy calcification). These findings may be related to the weakness of CCTA in the assessment of small vessels, such as the mid-distal portion of left circumflex artery and the diagonals, particularly in portions with the presence of large calcific plaques, where the beam-hardening artefacts might hinder the right residual lumen assessment. However, it is important to note that the concordance for the main and large vessels as left anterior descending artery and right coronary artery was also very high (94.2% and 87.5%, respectively) in patients with heavy calcific lesions. In summary, our study demonstrates that even in patients with heavy coronary calcifications, high atherosclerotic burden and diffuse CAD, in whom the appropriateness of CCTA is considered quite low, this non-invasive imaging tool showed to be suitable for treatment decision-making. This novel use of CCTA may be additional to the proven ability of the method to predict cardiac events in patients with non-obstructive and obstructive CAD and to provide a long-term warranty in clinically high-risk patients, including diabetics, in whom coronary arteries have been shown to be free from atherosclerotic disease [11, 12].

To further support the use of CCTA as a tool to provide interventionalists and cardiac surgeons with a non-invasive roadmap for myocardial revascularization, further innovative technologies, including new softwares addressing residual motion artefacts and beam-hardening artefacts [13], should be assessed and clinically validated. However, innovative image acquisition technologies, particularly when dual-energy instead of single-energy scanners are used, seem to be a more promising approach than new image reconstruction modalities. Dual-energy computed tomography (DECT) using calcium removal by material decomposition imaging has been proposed for improving the diagnostic performance of CCTA, particularly for the reduction of beam-hardening artefacts in patients with severe calcification [2]. This technique may be promising when used for the same purpose of the present study, i.e. treatment decision and procedural planning in patients with multivessel CAD. Indeed, in a recent study, Rodrıguez-Granillo *et al.* [14] showed that, compared to ICA, monochromatic imaging from DECT was able to identify a significantly larger atherosclerotic burden and a higher number of left main disease and severe proximal lesions. Moreover, DECT may also be useful in the identification of rupture-prone ‘vulnerable plaques’. Indeed, while calcified and non-calcified plaques may be easily diffentiated by single-energy CCTA, this technology faces a significant challenge in discriminaing the various anatomical components of non-calcified plaques. DECT may overcome these limitations, thus improving plaque characterization with a remarkable improvement in risk stratification and interventional procedure guidance [15]. A part from the anatomical assessment by CCTA, a potential implementation of the non-invasive management of three-vessel patients may be derived by the addition of a functional evaluation of coronary stenosis by the Fractional flow reserve derived from CTA (FFRCT), a non-invasive method able to identify lesion-specific ischaemia. In patients with multivessel CAD, FFRCT has shown to have good diagnostic performance with invasive pressure-wire assessment as reference (instantaneous flow reserve) in SYNTAX II trial [16, 17]. In particular, coronary CTA with FFRCT may provide to interventional cardiologists and cardiac surgeons a combined anatomical and functional non-invasive assessment of multivessel disease for the type and modality of revascularization. Indeed, one of the potential advantages of FFRCT is the possibility to interrogate the physiology of any segment in the epicardial coronary circulation [16]. Particularly, in patients undergoing CABG, the recognition of non-haemodynamically significant lesions may reduce unnecessary grafts and surgical time and might result in higher graft patency. A recent analysis of SYNTAX III trial showed that, by including the non-invasive functional evaluation with FFRCT, the heart teams changed treatment recommendation in 7% of the cases and modified the selection of vessels to be revascularized in 12% as compared to a CCTA assessment alone. Moreover, inclusion of FFRCT information on top of CCTA reduced from 92.3 to 78.8 the percentage of patients with haemodynamically significant three-vessel CAD [18].

### Limitations

Some limitations of this study should be acknowledged. First, although predefined in the protocol, the present study is a *post hoc* analysis of the SYNTAX III study. Second, the Agatston score was not calculated in the present study, which limits the comparability with previous CCTA studies regarding coronary calcium burden. Indeed a non-contrast scan of the chest was not acquired, to limit the radiation exposure. Moreover, in the present study, 53.2% of patients with three-vessel CAD did not present heavy calcifications. This rate may appear low in a setting of complex CAD. However, in 533 patients with three-vessel CAD undergoing CABG in the ACUITY trial, none-to-mild calcification based on angiography was observed in 289 patients (54.2%) [19]. Therefore, heavy calcifications could be observed in ∼50% for patients with three-vessel CAD, although the rates should be further evaluated. Third, the use of a cut-off value of 180° calcification to define the heavy calcific lesions, although proposed in a prospective multicentre trial on CCTA [1], remains subjective in separating patients in a calcified versus non-calcified group. Finally, because the evaluation of clinical outcome was not included in SYNTAX III trial, the clinical relevance of our findings needs to be confirmed by further prospective and randomized studies.

## CONCLUSIONS

In patients with three-vessel CAD with or without left main disease, despite heavy coronary calcifications affected in part the correlation between CCTA and ICA with regard to the anatomical SYNTAX score, the agreement on treatment decision was high and modestly influenced by the presence of heavily calcified lesions.

## SUPPLEMENTARY MATERIAL

Supplementary material is available at *ICVTS* online.

## Funding

The European Cardiovascular Research Institute sponsored this study with unrestricted research grants from General Electric Health Care and Heart Flow Inc.

**Conflict of interest:** Patrick W. Serruys reports consultancy fees from Abbott, Biosensors, Medtronic, Micell, Qualimed, Sinomedical Sciences, St. Jude Medical, Stentys, Svelte Medical Systems, Philips/Volcano, Xeltis, StentIt and HeartFlow. All other authors declared no conflict of interest.

### Author contributions

**Daniele Andreini:** Conceptualization; Data curation; Investigation; Supervision; Visualization; Writing—original draft; Writing—review & editing. **Kuniaki Takahashi:** Data curation; Formal analysis. **Saima Mushtaq:** Data curation; Methodology; Writing—review & editing. **Edoardo Conte:** Formal analysis; Investigation. **Rodrigo Modolo:** Data curation; Formal analysis. **Jeroen Sonck:** Data curation; Formal analysis; Validation; Writing—review & editing. **Johan De Mey:** Investigation; Methodology; Writing—review & editing. **Paolo Ravagnani:** Data curation; Investigation; Writing—review & editing. **Danny Schoors:** Data curation; Investigation. **Francesco Maisano:** Data curation; Formal analysis; Investigation. **Philipp Kaufmann:** Conceptualization; Data curation; Investigation. **Wietze Lindeboom:** Data curation; Formal analysis; Investigation. **Marie-angele Morel:** Methodology; Project administration; Resources. **Torsten Doenst:** Data curation; Formal analysis. **Ulf Teichgräber:** Data curation; Formal analysis; Validation. **Gianluca Pontone:** Formal analysis; Investigation; Methodology; Writing—review & editing. **Giulio Pompilio:** Formal analysis; Methodology. **Antonio Bartorelli:** Data curation; Writing—original draft; Writing—review & editing. **Yoshinobu Onuma:** Data curation; Formal analysis; Investigation; Methodology; Writing—review & editing. **Patrick W. Serruys:** Conceptualization; Data curation; Formal analysis; Funding acquisition; Supervision; Writing—review & editing.

### Reviewer information

Interactive CardioVascular and Thoracic Surgery thanks Daniyar Gilmanov, Ardawan J. Rastan and the other, anonymous reviewer(s) for their contribution to the peer review process of this article.

## Supplementary Material

ivab249_Supplementary_Data

## ABBREVIATIONS

CAD: Coronary artery disease
CCTA: Coronary computed tomography angiography
CI: Confidence interval
CTA: Computed tomography angiography
DECT: Dual-energy computed tomography
FFRCT: Fractional flow reserve derived from CTA
HT: Heart team
ICA: Invasive coronary angiography
PCI: Percutaneous coronary intervention

## References

[1] Vavere A , Arbab-Zadeh A, Rochitte CE, Dewey M, Ninuma H, Gottlieb I et al Coronary artery stenosis: accuracy of 64-detector row CT angiography in segments with mid, moderate, or severe calcification—a subanalysis of the CORE-64 trial. Radiology 2011;261:100–8. 21828192 10.1148/radiol.11110537 PMC3176425

[2] Andreini D , Pontone G, Mushtaq S, Bertella E, Conte E, Segurini C et al Diagnostic accuracy of rapid kilovolt peak-switching dual-energy CT coronary angiography in patients with a high calcium score. JACC Cardiovasc Imaging 2015;8:746–8. 25797129 10.1016/j.jcmg.2014.10.013

[3] Pontone G , Bertella E, Mushtaq S, Loguercio M, Cortinovis S, Baggiano A et al Coronary artery disease: diagnostic accuracy of CT coronary angiography—a comparison of high and standard spatial resolution scanning. Radiology 2014;271:688–94. 24520943 10.1148/radiol.13130909

[4] Collet C , Onuma Y, Andreini D, Sonck J, Pompilio G, Mushtaq S et al Coronary computed tomography angiography for heart team decision-making in multivessel coronary artery disease. Eur Heart J 2018;1:3689–98. 10.1093/eurheartj/ehy581 PMC6241466 30312411

[5] Cavalcante R , Onuma Y, Sotomi Y, Collet C, Thomsen B, Rogers C et al Non-invasive Heart Team assessment of multivessel coronary disease with coronary computed tomography angiography based on SYNTAX score II treatment recommendations: design and rationale of the randomised SYNTAX III Revolution trial. EuroIntervention 2017;12:2001–8. 27973335 10.4244/EIJ-D-16-00612

[6] Andreini D , Pontone G, Mushtaq S, Conte E, Perchinunno M, Guglielmo M et al Atrial fibrillation: diagnostic accuracy of coronary CT angiography performed with a whole-heart 230-µm spatial resolution CT scanner. Radiology 2017;284:676–84. 28445682 10.1148/radiol.2017161779

[7] Bland JM , Altman DG. Comparing methods of measurement: why plotting difference against standard method is misleading. Lancet 1995;346:1085–7. 7564793 10.1016/s0140-6736(95)91748-9

[8] Bablok W , Passing H, Bender R, Schneider B. A general regression procedure for method transformation. Application of linear regression procedures for method comparison studies in clinical chemistry, part III. J Clin Chem Clin Biochem 1988;26:783–90. 3235954 10.1515/cclm.1988.26.11.783

[9] Andreini D , Pontone G, Bartorelli AL, Agostoni P, Mushtaq S, Antonioli L et al Comparison of the diagnostic performance of 64-slice computed tomography coronary angiography in diabetic and non-diabetic patients with suspected coronary artery disease. Cardiovasc Diabetol 2010;9:80. 21114858 10.1186/1475-2840-9-80 PMC3006364

[10] Monizzi G , Sonck J, Nagumo S, Buytaert D, Van Hoe L, Grancini L et al Quantification of calcium burden by coronary CT angiography compared to optical coherence tomography. Int J Cardiovasc Imaging 2020;36:2393–402. 33205340 10.1007/s10554-020-01839-z

[11] Blanke P , Naoum C, Ahmadi A, Cheruvu C, Soon J, Arepalli C et al Long-term prognostic utility of coronary CT angiography in stable patients with diabetes mellitus. JACC Cardiovasc Imaging 2016;9:1280–8. 27568114 10.1016/j.jcmg.2015.12.027

[12] Andreini D , Pontone G, Mushtaq S, Bertella E, Conte E, Baggiano A et al Prognostic value of multidetector computed tomography coronary angiography in diabetes: excellent long-term prognosis in patients with normal coronary arteries. Diabetes Care 2013;36:1834–41. 23801796 10.2337/dc12-2123 PMC3687262

[13] Li P , Xu L, Yang L, Wang R, Hsieh J, Sun Z et al Blooming artifact reduction in coronary artery calcification by a new de-blooming algorithm: initial study. Sci Rep 2018;8:6945. 29720611 10.1038/s41598-018-25352-5 PMC5931966

[14] Rodrıguez-Granillo GA , Carrascosa P, Deviggiano A, Capunay C, de Zan MC, Goldsmit A. Extension y distribucion espacial de la carga ateroesclerotica mediante imagenes monocromaticas virtuales derivadas de tomografıa computarizada de doble energıa. Rev Esp Cardiol 2016;69:915–22. 27324434 10.1016/j.rec.2016.02.026

[15] Motoyama S , Ito H, Sarai M, Kondo T, Kawai H, Nagahara Y et al Plaque characterization by coronary computed tomography angiography and the likelihood of acute coronary events in mid-term follow-up. J Am Coll Cardiol 2015;66:337–46. 26205589 10.1016/j.jacc.2015.05.069

[16] Collet C , Miyazaki Y, Ryan N, Asano T, Tenekecioglu E, Sonck J et al Fractional flow reserve derived from computed tomographic angiography in patients with multivessel CAD. J Am Coll Cardiol 2018;71:2756–69. 29802016 10.1016/j.jacc.2018.02.053

[17] Collet Bortone CA. Expanding the Indications of Coronary Computed Tomography Angiography to Patients with Complex Coronary Artery Disease. 2020.

[18] Collet C, Sonck J, Leipsic J, Monizzi G, Buytaert D, Kitslaar P, Andreini D, De Bruyne B,. Implementing Coronary Computed Tomography Angiography in the Catheterization Laboratory. JACC Cardiovasc Imaging 2020:S1936-878X(20)30911-6. 10.1016/j.jcmg.2020.07.048 33248968

[19] Ertelt K , Généreux P, Mintz G, Reiss G, Kirtane A, Madhavan M et al Impact of the severity of coronary artery calcification on clinical events in patients undergoing coronary artery bypass grafting (from the Acute Catheterization and Urgent Intervention Triage Strategy Trial). Am J Cardiol 2013;112:1730–7. 24012035 10.1016/j.amjcard.2013.07.038

