# Supplementary material for: Impact of coronary calcification assessed by coronary CT angiography on treatment decision in patients with three-vessel CAD: insights from SYNTAX III trial
Source: Interact Cardiovasc Thorac Surg. 2021 Sep 20;34(2):176–84. doi: 10.1093/icvts/ivab249 (PMC8766208; doi:10.1093/icvts/ivab249)
Supplement: ivab249_Supplementary_Data [file ivab249_supplementary_data.docx]

**Supplementary Material**

**Table of contents**

|  |  | **Page** |
| --- | --- | --- |
| Table S1 | Coronary computed tomography angiography acquisition protocol | 2 |
| Table S2 | Baseline clinical characteristics. | 6 |
| Table S1. Coronary computed tomography angiography acquisition protocol | | |
| **Preparation**   - Assess heart rate and rhythm. Heart rate control (below 65 beats per minute) reduces motion artefacts. - Heart rate modulation for heart rate >60/min during breath holding.   - Oral: metoprolol tartrate 100 mg, one hour before the exam.  Atenolol 50 mg, one hour before the exam.   - IV: metoprolol 5 mg, repeated up to 5 times.   - Contraindications: conduction delays, hypotension, severe asthma, allergy to beta-blockers, reduced left ventricle ejection fraction.   - Consider ivabradine for patients with contra-indications to beta-blockers (in case of ivabradine the dose suggested is 5 mg twice a day for at least 3-4 days before the scan). - Full explanation of exam, and practice breath hold. Ensure breath hold time will be sufficient for scan time. Evaluate impact of breath holds on heart rate. - **Nitrates and FFR_CT_**   • use NTG preferably 3 minutes prior to CT image acquisition.  • use 1-2 sprays (0.4mg-0.8mg).  • use beta-blocker with it to avoid reflex tachycardia/vasoconstriction.  • additional Beta blockade may be given after nitroglycerin to counteract the reflex tachycardia.   - Confirm absence of allergy to contrast media (consider prophylaxis for patients with doubtful or mild reactions to contrast in the past). | | |
| **Patient installation**   - Attach ECG leads, avoid respiratory muscles, and check signal stability during breath hold. - Placement of an IV catheter that allows a flow of at least 5 ml/sec   **Data acquisition:**   1. Overview/scout of the entire chest. 2. Contrast enhancement:    - ≥300 g/L iodine contrast medium.    - Injection rate: 5-6 ml/s.    - Total amount depends on the patient size, the scan mode and the scan duration.    - Contrast-scan timing:      - ***Test/Timing Bolus***: 15-20 ml of contrast is injected, preferably followed by a saline flush. Place the localizer line one centimeter below the carina and just above the base of the heart, the optimal location to find the ascending aorta for a timed contrast injection. The time of (maximum) enhancement is used as the delay of the data acquisition after start of contrast injection.      - *Bolus tracking/Smart Prep*: arrival of the (entire) bolus is monitored by using a 4-chamber view.    - A saline bolus of ≈50 ml is injected after the contrast medium at the same rate. 3. Scan mode :  - ECG-triggered one-beat scan mode should be used. For HR <65, 75% of the R-R cycle is appropriate. For HR>65 or variable heart rates, 40-80% of the R-R cycle is appropriate with ECG mA modulation. Consider use of Auto-Gating functionality on the system.  1. Acquisition parameters:  - Thinnest detector width. - For patients acquired in standard mode we suggest 100 kVp/500 mA for BMI<25; 100 kVp/550 mA for BMI included between 25 and 30 and 120 kVp/600 mA for BMI>30; for HD mode we suggest 100 kVp for BMI<25, together with 550 mA - Scan range: from 1-2 cm below the carina until the caudal border of the heart. - High Definition Mode should be used preferably **except** in patients with BMI > 25   Alternate Data Acquisition protocols may be applicable based on local experience and expertise. These alternative protocols will be reviewed and approved by the Steering Committee including potential review of sample clinical cases. | | |
| **Image reconstruction (appropriately labelled):**   - 0.625mm slice thickness. - ASIR-V 50% in all cases should be provided. Additional ASIR-V levels may be provided if ASIR-V 50% is inadequate. - Field-of-view enclosing the **entire heart** (cover inferior carina to lower heart border) (approx. 18 x 18 cm). - Standard kernel reconstructions of at least **three** different phases. Depending on the scan protocol both diastolic and systolic reconstructions should be performed. - Reconstructions should be optimized for the segments of interest (ROI). In case of suboptimal image quality, other phases should be explored. - Additional high-definition reconstructions should be provided at the optimal phase(s). If High Definition mode was not performed, then Detail kernel reconstructions should be provided. - If motion artefacts persist in the optimal phase images, the standard and high definition (or detail) reconstructions should be done with “Temporal Enhanced” enabled and SnapShot. Freeze processing should be performed on the Advantage Workstation. | | |
| **DVD/USB recording:**   - Scout images. - ECG trace. - Standard kernel reconstructions for at least one (or the same) optimal phase for each diseased coronary segment, preferably three or more datasets including both systolic and diastolic phases. SnapShot Freeze processed images should be provided if any motion persists in the optimal phases (the accuracy of FFR-CT need to be evaluated for images reconstructed with SSF). - HD or Detail reconstructions for at least one (or the same) optimal phase for each diseased segment. SnapShot Freeze processed images should be provided if any motion persists in the optimal phases (the accuracy of FFR-CT need to be evaluated for images reconstructed with SSF). | | |

**Table S2. Baseline clinical characteristics.**

| **Characteristics** | **N=223 patients** |
| --- | --- |
| **Demographics** |  |
| Age (years; mean±SD) | 67.6±8.9 |
| Male (%, n) | 84.3 (188/223) |
| **CAD risk factors** |  |
| Current smoking (%, n) | 22.6 (48/212) |
| Diabetes mellitus (%, n) | 37.7 (84/223) |
| Treatment for diabetes (%, n) |  |
| Insulin (%, n) | 10.3 (23/223) |
| Medication (%, n) | 25.6 (57/223) |
| Diet (%, n) | 0.9 (2/223) |
| Hypertension (%, n) | 74.9 (167/223) |
| Hyperlipidemia (%, n) | 70.0 (154/220) |
| Family history of CAD (%, n) | 35.6 (67/188) |
| **Medical history** |  |
| Previous stroke (%, n) | 8.1 (18/223) |
| Previous myocardial infarction (%, n) | 0.9 (2/220) |
| COPD (%, n) | 13.0 (29/223) |
| PVD (%, n) | 17.5 (39/223) |
| **Clinical presentation** |  |
| Silent ischemia (%, n) | 42.2 (94/223) |
| Stable angina (%, n) | 48.9 (109/223) |
| CCS class (%, n) |  |
| I (%, n) | 15.6 (17/109) |
| II (%, n) | 56.0 (61/109) |
| III (%, n) | 25.7 (28/109) |
| IV (%, n) | 2.8 (3/109) |
| Unstable angina (%, n) | 9.0 (20/223) |
| BMI (kg/m2, mean±SD) | 26.5±3.7 |
| Creatinine clearance (ml/min, mean±SD) | 81.6±27.5 |
| LVEF (%, mean±SD) | 54.6±11.0 |
| Heart rate during CCTA acquisition (mean±SD) | 54.6±11.0 |

*CAD= coronary artery disease; COPD= chronic obstructive pulmonary disease; PVD= peripheral vascular disease; BMI= body mass index; LEVF= left ventricle ejection fraction; CCTA= coronary computed tomography angiography.*
